# The COVID-19 pandemic and its psychological impact on tunisian health professionals

**DOI:** 10.1192/j.eurpsy.2021.773

**Published:** 2021-08-13

**Authors:** L. Aribi, R. Jbir, N. Messedi, O. Bouattour, N. Kolsi, Z. Kallel, F. Charfeddine, J. Aloulou

**Affiliations:** 1 Psychiatry ‘b’, University Hospital Hedi Chaker Sfax, Sfax, Tunisia; 2 Psychiatry ‘b’, university hospital Hedi chaker Sfax, sfax, Tunisia; 3 Psychiatry, Hedi chaker Sfax, sfax, Tunisia

**Keywords:** COVID-19, health professionals, Post traumatic Stress Disorder, Anxiety

## Abstract

**Introduction:**

In March 2020,World Health Organization characterized the COVID-19 outbreak as pandemic. This new health situation has created an anxiety-provoking climate among health professionals

**Objectives:**

To provide risks associated with the exposure of caregivers to COVID-19 for their mental health by studying the prevalence and predictors of post-traumatic stress disorder, anxiety and depression.

**Methods:**

Our study was descriptive and analytical cross-sectional, carried out with healthcare in different hospitals in Tunisia, between 13 May until 20 June 2020. An online survey was sent to caregivers. mental health was assessed using 3 scales: PCL-5:measure the symptoms of post-traumatic stress disorder HADS :screen for anxiety and depression. PSQI:see the existence of a disturbance in sleep quality

**Results:**

125 caregivers participated in the survey. The average age was 32 years. The participants were predominantly female (72.8%), married (48%), and had at least one child (39.2%). 28.7% of participants had increased their consumption of coffee, especially anxious people (p = 0.001). Anxiety was retained in 44% and depression in 47.2%. Anxiety was significantly related to sex with (p = 0.039) and affects more women. The consumers of coffee developed more anxiety (p = 0.034) and depression (p = 0.026). 42.4% of participants had presented post-traumatic stress disorder. Three parameters were correlated with post-traumatic stress disorder: young age, having children and fewer years of professional experience. 62.4% of caregivers had a bad quality of sleep

**Conclusions:**

This health crisis had a major impact on the mental health of our heroes. So, we should provide for them with the necessary support.

